# Perioperative goal-directed therapy and postoperative complications in different kind of surgical procedures: an updated meta-analysis

**DOI:** 10.1186/s44158-021-00026-3

**Published:** 2021-12-15

**Authors:** Mariateresa Giglio, Giandomenico Biancofiore, Alberto Corriero, Stefano Romagnoli, Luigi Tritapepe, Nicola Brienza, Filomena Puntillo

**Affiliations:** 1 Anesthesia and Intensive Care Unit, Policlinico di Bari, Piazza G. Cesare, 11, 70124 Bari, Italy; 2 UO Anestesia e Rianimazione Trapianti, Università degli Studi, Pisa, Italy; 3 Anesthesia and Intensive Care Unit, Department of Emergency and Organ Transplantation, University of Bari, Bari, Italy; 4 Anesthesia, Intensive Care Unit and Pain Unit, Department of Interdisciplinary Medicine, University of Bari, Bari, Italy; 5 Dipartimento di Anestesia e Rianimazione, Azienda Ospedaliero-Universitaria Careggi, Firenze, Italy; 6 Direttore UOC Anestesia e Rianimazione, AO San Camillo Forlanini-Roma, Rome, Italy

**Keywords:** Postoperative complications, Fluid therapy, Cardiac output, Meta-analysis

## Abstract

**Background:**

Goal-directed therapy (GDT) aims to assure tissue perfusion, by optimizing doses and timing of fluids, inotropes, and vasopressors, through monitoring of cardiac output and other basic hemodynamic parameters. Several meta-analyses confirm that GDT can reduce postoperative complications. However, all recent evidences focused on high-risk patients and on major abdominal surgery.

**Objectives:**

The aim of the present meta-analysis is to investigate the effect of GDT on postoperative complications (defined as number of patients with a least one postoperative complication) in different kind of surgical procedures.

**Data sources:**

Randomized controlled trials (RCTs) on perioperative GDT in adult surgical patients were included. The primary outcome measure was complications, defined as number of patients with at least one postoperative complication. A subgroup-analysis was performed considering the kind of surgery: major abdominal (including also major vascular), only vascular, only orthopedic surgery. and so on.

**Study appraisal and synthesis methods:**

Meta-analytic techniques (analysis software RevMan, version 5.3.5, Cochrane Collaboration, Oxford, England, UK) were used to combine studies using odds ratios (ORs) and 95% confidence intervals (CIs).

**Results:**

In 52 RCTs, 6325 patients were enrolled. Of these, 3162 were randomized to perioperative GDT and 3153 were randomized to control. In the overall population, 2836 patients developed at least one complication: 1278 (40%) were randomized to perioperative GDT, and 1558 (49%) were randomized to control. Pooled OR was 0.60 and 95% CI was 0.49–0.72. The sensitivity analysis confirmed the main result.

The analysis enrolling major abdominal patients showed a significant result (OR 0.72, 95% CI 0.59–0.87, *p* = 0.0007, 31 RCTs, 4203 patients), both in high- and low-risk patients. A significant effect was observed in those RCTs enrolling exclusively orthopedic procedures (OR 0.53, 95% CI 0.35–0.80, *p* = 0.002, 7 RCTs, 650 patients. Also neurosurgical procedures seemed to benefit from GDT (OR 0.40, 95% CI 0.21–0.78, *p* = 0.008, 2 RCTs, 208 patients). In both major abdominal and orthopedic surgery, a strategy adopting fluids and inotropes yielded significant results. The total volume of fluid was not significantly different between the GDT and the control group.

**Conclusions and implications of key findings:**

The present meta-analysis, within the limits of the existing data, the clinical and statistical heterogeneity, suggests that GDT can reduce postoperative complication rate. Moreover, the beneficial effect of GDT on postoperative morbidity is significant on major abdominal, orthopedic and neurosurgical procedures. Several well-designed RCTs are needed to further explore the effect of GDT in different kind of surgeries.

**Supplementary Information:**

The online version contains supplementary material available at 10.1186/s44158-021-00026-3.

## Background

Goal-directed therapy (GDT) is a strategy that aims to optimize dose and timing of fluids, inotropes, and vasopressors, through monitoring of cardiac output and other basic hemodynamic parameters, in order to assure an adequate tissue perfusion and oxygen delivery. In the last 30 years, many authors have reported that GDT adoption can reduce the incidence of morbidity, and in some studies, mortality [1–3]. Several meta-analyses [1, 4] support its use in high-risk patients, and a recent trial reports a significant effect also in low–moderate-risk patients [5]. However, all recent meta-analyses focused mainly on major abdominal surgery and on high-risk patients [6–8], while the evidence is less clear in other surgical procedures.

The aim of the present updated meta-analysis is to investigate the effect of GDT on postoperative complications in different kind of surgical procedures. Moreover, we analyzed the amount of crystalloids and colloids administered during the intraoperative period in order to verify if a GDT approach is useful to control the total amount of administered fluids.

## Methods

### Eligibility criteria

RCTs were selected according to the following inclusion criteria [9]:

- Types of participants. Adult patients (ages 18 years and older) undergoing major non cardiac surgery were considered. Studies involving mixed populations of critically ill, nonsurgical patients, or postoperative patients with sepsis or organ failure were excluded.
- Types of interventions. GDT was defined as monitoring and manipulation of hemodynamic parameters to reach normal or supranormal values by fluid infusion alone or in combination with inotropic therapy in the perioperative period within 8 h after surgery. Studies including late hemodynamic optimization treatment were excluded.
- Types of comparisons. Trials comparing the beneficial and harmful effects of GDT versus standard hemodynamic therapy were considered. RCTs with no description or no difference in optimization strategies between groups, as well as RCTs in which therapy was titrated to the same goal in both groups or was not titrated to predefined end-points were excluded.
- Types of outcome measures. The primary outcome measure was complications, defined as number of patients with at least one postoperative complication. Postoperative complications include minor and major cardiac, renal, gastrointestinal, infective and respiratory ones. Mortality was not included. Sensitivity analysis was planned including only low risk of bias trials (see below). Studies were splitted considering the kind of surgery (i.e., major abdominal, orthopedic, vascular, and so on). Moreover, studies were divided on the basis of the strategy adopted (i.e., only fluids or fluids and inotropes). In those studies that used fluids alone, the volume of crystalloids and of colloids, as well as the total volume of fluids received during the GDT period were also analyzed.
- Types of studies. RCTs on perioperative GDT in surgical patients were included. No language, publication date, or publication status restrictions were imposed.

### Information sources

Different search strategies (last update July 2021) were performed to retrieve relevant randomized controlled trials (RCTs) by using MEDLINE, The Cochrane Library and EMBASE databases. No date restriction was applied for MEDLINE and The Cochrane Library databases, while the search was limited to 2008–2021 for EMBASE database [10]. Additional RCTs were searched in The Cochrane Library and the Database of Abstracts of Reviews of Effects (DARE) databases and in the reference lists of previously published reviews and retrieved articles. Other data sources were hand-searched in the annual proceedings (2008–2020) of the Society of Critical Care Medicine, the European Society of Intensive Care Medicine, the Society of Cardiovascular Anesthesiologists, the Royal College of Anaesthetists, the American Society of Anesthesiologists. In order to reduce publication bias, abstracts were searched [11]. Publication language was not a search criterion.

### Search terms

Trials selection was performed by using the following search terms: randomized controlled trial, controlled clinical trial, surgery, goal-directed, goal oriented, goal target, cardiac output, cardiac index, DO_2_, oxygen consumption, cardiac volume, stroke volume, fluid therapy, fluid loading, fluid administration, optimisation, optimization, supranormal. The search strategies used for the MEDLINE, The Cochrane Library, and EMBASE databases are reported in Supplementary material 1.

### Study selection

Two investigators (FP, LT) examined at first each title and abstract to exclude clearly irrelevant studies and to identify potentially relevant articles. Other two investigators (MG, NB) independently determined eligibility of full-text articles retrieved. The names of the author, institution, journal of publication and results were unknown to the two investigators at this time.

### Data abstraction and study characteristics

Data were independently collected by two investigators (GB, SR), with any discrepancy resolved by re-inspection of the original article. To avoid transcription errors, the data were input into statistical software and rechecked by different investigators (AC, NB).

### RCT data gathered

Data abstraction included surgical risk (defined by the authors on the basis of POSSUM score [12], ASA physical status classification, age > 60 years, pre-operative morbidity, and type of surgery), type of surgery (i.e., elective or emergent, abdominal, thoracic, vascular), anesthesiological management, and hemodynamic goal-directed therapy (end-points, therapeutic intervention, and monitoring tools). The volume of crystalloids and of colloids, as well as the total volume of fluid received during the GDT period was also analyzed.

### Risk of bias in individual studies

A domain-based evaluation, as proposed by the Cochrane Collaboration, was used to evaluate the methodological quality of RCTs [13]. This is a two-part tool, addressing seven specific domains that are strongly associated with bias reduction [14, 15]. Each domain in the tool includes one or more specific entries in a ‘risk of bias’ table. Within each entry, the first part of the tool describes what was reported to have happened in the study, in sufficient detail to support a judgement about the risk of bias. The second part of the tool assigns a judgement relating to the risk of bias for that entry. This is achieved by assigning a judgement of ‘low risk’, ‘high risk’, or ‘unclear risk’ of bias. After each domain was completed, a ‘risk of bias summary’ figure presenting all of the judgements in a cross-tabulation of study by entry is generated. The green plus indicates low risk of bias, the red minus indicates high risk of bias, the white color indicates unclear risk of bias. For each study, the number of green plus obtained for every domain was calculated: RCTs with 5 or 6 green plus were considered as having an overall low risk of bias.

### Summary measures and planned method of analysis

Meta-analytic techniques (analysis software RevMan, version 5.3.5, Cochrane Collaboration, Oxford, England, UK) were used to combine studies using odds ratios (ORs) and 95% confidence intervals (CIs) for dichotomous variables, and weighted mean difference (WMD) and 95% CI for continuous variables. A statistical difference between groups was considered to occur if the pooled 95% CI did not include 1 for the OR. An OR less than 1 favored GDT when compared with control group. Two-sided *p* values were calculated. A random-effects model was chosen for all analyses. Statistical heterogeneity and inconsistency were assessed by using the *Q* and *I*^2^ tests, respectively [16, 17]. When the *p* value of the *Q* test was < 0.10 and/or the *I*^2^ was > 40%, heterogeneity and inconsistency were considered significant [18].

## Results

### Study selection

**Fig. 1:**
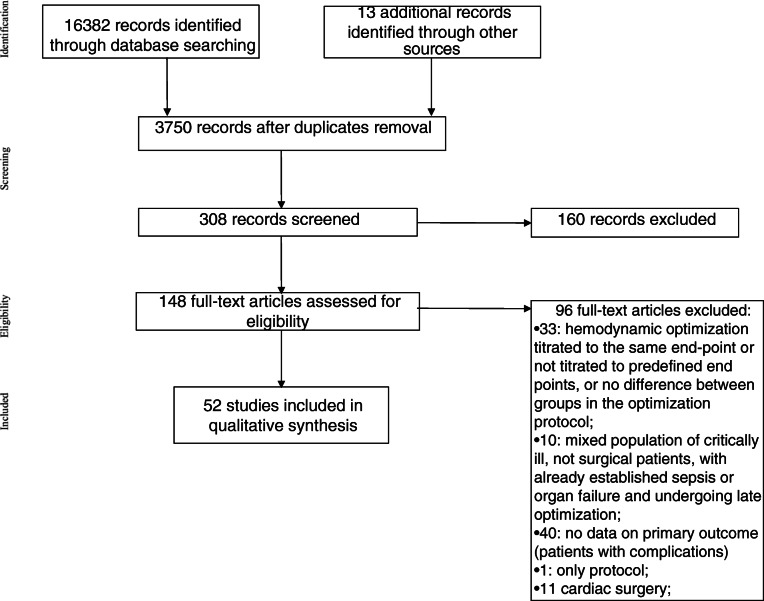
Flow chart summarizing the studies selection procedure for the meta-analysis. RCT, andomized controlled trial

The search strategies identified 3561 (MEDLINE), 10306 (Cochrane Library), and 3110 (EMBASE) articles. Thirteen articles were identified through other sources (congress abstracts, reference lists). After initial screening and subsequent selection, a pool of 148 potentially relevant RCTs was identified. The subsequent eligibility process (Fig. 1) excluded 96 articles and, therefore, 52 articles (5, 18–68) with a total sample of 6315 patients, were considered for the analysis.

### Study characteristics

All inclueded articles evaluated the effects of hemodynamic optimization on morbidity as primary or secondary outcome and had a population sample of adult surgical patients, undergoing both elective and emergent procedures (Table 1). The studies were performed in Australia, the USA, Europe, Canada, Brazil, China, India, and Japan from 1991 to 2021 (Table 1) and were all published in English.

**Table 1:** Characteristics of included studies

Author, year, country	Surgery	Goal-directed therapy (tools and goals)	Modality of optimization
**Ackland et al.** **[**19**]** 2015, Europe	Major elective abdominal surgery	Lidco plus; SV < 10%, DO_2_ > 600 L∙min^−1^∙m^−2^	Fluids and inotropes
**Arslan-Carlon et al.** **[**20**]** 2020, USA	Open radical cystecctomy	FloTrac/Vigileo; SV < 10% CI ≥ 2.5 L min^−1^ m^−2^	Fluids
**Bahlmann et al.** **[**21**]** 2019, Europe	Tranthoracic eophageal resection	FloTrac/Vigileo; SV < 10% CI ≥ 2.5 L min^−1^ m^−2^	Fluids and inotropes
**Bartha et al.** **[**22**]** 2018, Europe	Orthopaedic	Lidco; SV < 10%, DO_2_ > 600 L min^−1^ m^−2^	Fluids and inotropes
**Bender et al.** **[**23**]** 1997, USA	Elective aortic and vascular	PAC; CI ≥ 2.8 L min^−1^ m^−2^, 8 ≤ Pcwp ≤ 14 mmHg, SVR ≤ 1100 dyne s cm^−5^	Fluids and inotropes
**Benes et al.** **[**24**]** 2010, Europe	Elective abdominal	FloTrac/Vigileo; CI ≥ 2.5 L min^−1^ m^−2^	Fluids and inotropes
**Bisgaard et al.** **[**25**]** 2013, Europe	Elective peripheral vascular	Lidco; SV < 10%, DO_2_ > 600 L min^−1^ m^−2^	Fluids and inotropes
**Brandstrup et al.** **[**26**]** 2012, Europe	Elective abdominal	Esophageal Doppler SV increase > 10%	Fluids
**Broch et al.** **[**27**]** 2016, Europe	Major abdominal	Nexfin system; PPV > 10% CI ≥ 2.5 L min^−1^ m^−2^	Fluids and inotropes
**Calvo Vecino et al.** **[**5**]** 2018, Spain	Major abdominal, urological, gynaecological, or orthopedic surgery	(CardioQ, EDM; SV increase > 10% CI ≥ 2.5 L min^−1^ m^−2^	Fluids and inotropes
**Cecconi et al.** **[**28**]** 2011, Europe	Orthopaedic	FloTrac/Vigileo; SV < 10%, DO_2_ > 600 L min^−1^ m^−2^	Fluids and inotropes
**Challand et al.** **[**29**]** 2013, Europe	Major abdominal	Esophageal Doppler SV increase of 10%	Fluids
**Colantonio et al.** **[**30**]** 2015, Europe	Cytoreductive surgery	FloTrac/Vigileo; CI ≥ 2.5 L min^−1^ m^−2^ SVI > 35 ml∙min^−1^∙m^−2^	Fluids and inotropes
**Correa-Gallego et al.** **[**31**]** 2015, Europe	Elective liver resection	FloTrac/Vigileo; SVV < 2 DS of pre-induction	Fluids
**Elgendy et al.** **[**32**]** 2017, Africa	Major abdominal	FloTrac/Vigileo; SVV < 12%, CI ≥ 2.5 L min^−1^ m^−2^	Fluids and inotropes
**Forget et al.** **[**33**]** 2011, Europe	Major abdominal	Masimo set pulse oxymeter; PVI < 13%	Fluids
**Gomez-Izquierdo et al.** **[**34**]** 2017, Canada	Colorectal surgery	Cardio Q rise of SV > 10%	Fluids
**Jammer et al.** **[**35**]** 2010, Europe	Colo-rectal surgery	CVC ScVO_2_ > 75%	Fluids
**Jhanii et al.** **[**36**]** 2010, Europe	Elective gastro-intestinal	Not stated rise of SV > 10%	Fluids and inotropes
**Joosten et al.** **[**37**]** 2019, Europe	Major abdomina	Clearsight closed loop; SVV > 13%, CI 2.5 3 L min^−1^ m^−2^	Fluids
**Kaufmann et al.** **[**38**]** 2018, Europe	Orthopaedic	Esophageal Doppler rise of SV > 10% CI ≥ 2.5 L min^−1^ m^−2^	Fluids and inotropes
**Kumar et al.** **[**39**]** 2016, India	Elective abdominal	FloTrac/Vigileo; SVV < 10%,	Fluids and inotropes
**Lobo et al.** **[**40**]** 2000, Brazil	Elective major abdominal or vascular	PAC; DO_2_ > 600 mL min^−1^ m^−2^	Fluids and inotropes
**Lopes et al.** **[**41**]** 2007**,** Brazil	Elective abdominal	Radial artery line; ΔPP ≤ 10%	Fluids
**Luo et al.** **[**42**]** 2017, China	Neurosurgery	FloTrac/Vigileo; SVV < 15%, CI ≥ 2.5 L min^−1^ m^−2^	Fluids and inotropes
**Mayer et al.** **[**43**]** 2010, Europe	Major abdominal	FloTrac/Vigileo; CI ≥ 2.5 L min^−1^ m^−2^	Fluids and inotropes
**Mikor et al.** **[**44**]** 2015, Europe	Major abdominal	Cevox ScVO2 > 75% or reduction 3%	Fluids and inotropes
**Moppett et al.** **[**45**]** 2014, Europe	Emergent orthopaedic	LiDCO; SV increase < 10%	Fluids
**Mukai et al.** **[**46**]** 2020, Japan	Tranthoracic eophageal resection	FloTrac/Vigileo; SVV < 12%,	Fluids and inotropes
**Noblett et al.** **[**47**]** 2005, Europe	Major abdominal	Esophageal Doppler; SV optimization	Fluids
**Pearse et al.** **[**48**]** 2005, Europe	Elective or emergent major general	LiDCO; DO_2_ > 600 mL min^−1^ m^−2^, SV > 10%	Fluids and inotropes
**Pearse et al.** **[**49**]** 2014, Europe	Major general	LiDCO; SV increase < 10%	Fluids and inotropes
**Pestana et al.** **[**50**]** 2014, multicentric	Major abdominal	NICOM; CI ≥ 2.5 L min^−1^ m^−2^	Fluids and inotropes
**Pillai et al.** **[**51**]** 2011 USA	Radical cystectomy	Cardio Q increase of SV > 10%	Fluids
**Salzwedel et al.** **[**52**]** 2013, Europe	Major abdominal	ProAQT PPV > 10% CI ≥ 2.5 L/min/m^2^	Fluids and inotropes
**Schereen et al.** **[**53**]** 2013, Europe	Major abdominal and urologic	FloTrac/Vigileo; SVV < 10%	Fluids
**Schmid et al.** **[**54**]** 2019, Europe	Orthopaedic	PulsioFlex SVI increase < 10% CI ≥ 2.5 L/min/m^2^	Fluids and inotropes
**Shoemaker et al.** **[**55**]** 1998, USA	Emergent or elective major abdominal (general or vascular)	PAC; CI > 4.5 L min^−1^ m^−2^, DO_2_ > 600 mL min^−1^ m^2^, VO_2_ > 170 mL min^−1^ m^−2^	Fluids and inotropes
**Sinclair et al.** **[**56**]** 1997, Europe	Orthopaedic	Esophageal Doppler SV optimization with FTc between 0.35–0.4 s	Fluids
**Srinvasa et al.** **[**57**]** 2012, Australia	Elective colectomy	Esophageal Doppler SV optimization with FTc between 0.35–0.4 s	Fluids
**Stens et al.** **[**58**]** 2017, Europe	Major abdominal	Nexfin device PPV < 12% CI > 2.5 L min^−1^ m^−2^	Fluids and inotropes
**Szturz et al.** **[**59**]** 2019, Europe	Major abdominal	Esophageal Doppler FTc < 330 ms CI > 2.5 L min^−1^ m^−2^	Fluids and inotropes
**Ueno et al.** **[**60**]** 1998, China	Hepatic resection	PAC; CI > 4.5 L min^−1^ m^−2^, DO_2_ > 600 mL min^−1^ m^2^, VO_2_ > 170 mL min^−1^ m^−2^	Fluids and inotropes
**Van Beest** **[**61**]** 2014, Europe	Elective major	In spectra system StO2 > 80%	Fluids and inotropes
**Venn et al.** **[**62**]** 2002, Europe	Orthopaedic	Esophageal Doppler SV optimization with FTc > 0.4 s	Fluids
**Wakeling et al.** **[**63**]** 2005, Europe	Elective major bowel	Esophageal Doppler; SV optimization and rise in CVP < 3 mmHg	Fluids
**Weineberg et al.** **[**64**]** 2017, Australia	Pancreaticoduodenectomy	FloTrac/Vigileo; SVV < 20% baseline CI ≥ 2 L min^−1^ m^−2^	Fluids and inotropes
**Weineberg et al.** **[**65**]** 2019, Australia	Liver resection	FloTrac/Vigileo; SVV< 20% baseline CI ≥ 2.2 L min^−1^ m^−2^	Fluids and inotropes
**Wilson et al.** **[**66**]** 1999, Europe	Elective major (abdominal, vascular, urologic)	PAC; DO_2_ > 600 mL min^−1^ m^−2^	Fluids and inotropes
**Wu et al.** **[**67**]** 2017, China	Neurosurgery	FloTrac/Vigileo; SVV < 12%, CI > 2.5 L min^−1^ m^−2^	Fluids and inotropes
**Zhang et al.** **[**68**]** 2013, China	Thorascopic lobectomy	FloTrac/Vigileo; SVV < 10%, CI > 2.5 L min^−1^ m^−2^	Fluids and inotropes
**Zheng et al.** **[**69**]** 2013, China	Elective abdominal	FloTrac/Vigileo; SVI > 35 mL/m^2^, CI ≥ 2.5 L min^−1^ m^−2^	Fluids and inotropes

*Abbreviations*: *PPV* pulse pressure variation, *PVI* Pleth Variability Index, *SVV* stroke volume variation, *SV* stroke volume, *CI* cardiac index, *CVP* central venous pressure, *SVI* stroke volume index, *SVR* systemic vascular resistance, *ScvO*_*2*_ central venous oxygen saturation, *DO*_*2*_ oxygen delivery, *Pcwp* pulmonary capillary wedge pressure, *PAC* pulmonary artery catheter, *FTc* flow-time-corrected, *VO*_*2*_ oxygen consumption, *LiDCO* lithium dilution cardiac output monitoring, *NICOM* non-invasive cardiac output monitoring obtained via bioreactance, *CVC* central venois catheter, *StO2* tissue oxygenation, *DS* standard deviation, *ΔPP* variation of arterial pressure

Data concerning population and type of surgery are presented in Table 1. The risk of bias assessment for each trial is showed in Table 2.

**Table 2:** The risk of bias assessment for each trial, according to the Cochrane domain-based evaluation

Author, year, country	Blinding of participants and personnel (performance bias)	Random sequence generation (selection bias)	Allocation concealment (selection bias)	Outcome assessment (detection bias)	Incomplete outcome (attrition bias)	Selective reporting (reporting bias)
**Ackland et al** ^**19**^ 2015, Europe	**+**	**+**		**+**	**+**	**+**
**Arslan-Carlon et al.** **[**20**]** 2020, USA	**+**	**+**	**+**	**+**	**+**	**+**
**Bahlmann et al.** **[**21**]** 2019, Europe	**+**	**+**	**+**	**+**	**+**	**+**
**Bartha et al.** **[**22**]** 2018, Europe		**+**	**+**	**+**	**+**	**+**
**Bender et al.** **[**23**]** 1997, USA	**−**	**−**	**−**		**−**	
**Benes et al.** **[**24**]** 2010, Europe		**+**	**+**	**+**	**+**	**+**
**Bisgaard et al.** **[**25**]** 2013, Europe	**+**	**+**		**+**	**+**	**+**
**Brandstrup et al.** **[**26**]** 2012, Europe	**+**	**+**	**+**	**+**	**+**	**+**
**Broch et al.** **[**27**]** 2016, Europe		**+**			**+**	**+**
**Calvo Vecino et al.** **[**5**]** 2018, Spain	**+**	**+**	**+**	**+**	**+**	**+**
**Cecconi et al.** **[**28**]** 2011, Europe			**+**	**+**	**+**	**+**
**Challand et al.** **[**29**]** 2013, Europe	**+**	**+**	**+**	**+**		**+**
**Colantonio et al.** **[**30**]** 2015, Europe	**+**	**+**		**+**	**+**	**+**
**Correa-Gallego et al.** **[**31**]** 2015, Europe	**+**	**+**	**+**	**+**	**+**	
**Elgendy et al.** **[**32**]** 2017, Africa	**+**	**−**		**+**		**+**
**Forget et al.** **[**33**]** 2011, Europe		**+**	**+**	**+**	**+**	**+**
**Gomez-Izquierdo et al.** **[**34**]** 2017, Canada	**+**	**+**	**+**	**+**	**+**	**+**
**Jammer et al.** **[**35**]** 2010, Europe		**+**	**+**	**+**	**+**	**+**
**Jhanii et al.** **[**36**]** 2010, Europe		**+**	**+**	**+**	**+**	**+**
**Joosten et al.** **[**37**]** 2019, Europe	**+**	**+**	**+**		**+**	**+**
**Kaufmann et al.** **[**38**]** 2018, Europe	**+**	**+**	**+**	**+**	**+**	
**Kumar et al.** **[**39**]** 2016, India		**−**	**+**	**+**	**+**	**+**
**Lobo et al.** **[**40**]** 2000, Brazil		**+**			**+**	**+**
**Lopes et al.** **[**41**]** 2007**,** Brazil	**−**	**−**	**+**	**+**	**+**	
**Luo et al.** **[**42**]** 2017, China	**−**	**−**	**+**	**−**		
**Mayer et al.** **[**43**]** 2010, Europe			**+**	**+**	**+**	**+**
**Mikor et al.** **[**44**]** 2015, Europe	**+**	**+**		**+**	**+**	**+**
**Moppett et al.** **[**45**]** 2014, Europe	**+**	**+**	**+**	**+**	**+**	**+**
**Mukai et al.** **[**46**]** 2020, Japan	**+**	**+**			**+**	**+**
**Noblett et al.** **[**47**]** 2005, Europe	**+**	**−**	**+**	**+**	**+**	**+**
**Pearse et al.** **[**48**]** 2005, Europe		**+**	**+**	**+**	**+**	**+**
**Pearse et al.** **[**49**]** 2014, Europe	**+**	**+**	**+**	**+**	**+**	**+**
**Pestana et al.** **[**50**]** 2014, multicentric	**+**	**+**	**+**	**+**	**+**	
**Pillai et al.** **[**51**]** 2011 USA	**−**	**−**	**−**	**−**		
**Salzwedel et al.** **[**52**]** 2013, Europe	**+**	**+**	**+**	**+**	**+**	**+**
**Schereen et al.** **[**53**]** 2013, Europe			**+**	**+**	**+**	**+**
**Schmid et al.** **[**54**]** 2019, Europe	**+**	**+**	**+**	**+**	**+**	
**Shoemaker et al.** **[**55**]** 1998, USA	**−**	**−**	**−**	**−**	**−**	**+**
**Sinclair et al.** **[**56**]** 1997, Europe	**+**		**+**	**+**	**+**	**+**
**Srinvasa et al.** **[**57**]** 2012, Australia	**+**	**+**	**+**	**+**	**+**	
**Stens et al.** **[**58**]** 2017, Europe		**+**	**+**			**+**
**Szturz et al.** **[**59**]** 2019, Europe	**+**	**+**	**+**	**+**	**+**	
**Ueno et al.** **[**60**]** 1998, China	**−**	**+**	**−**			
**Van Beest** **[**61**]** 2014, Europe	**−**	**−**	**−**	**+**	**+**	**+**
**Venn et al.** **[**62**]** 2002, Europe		**+**	**+**	**+**	**+**	**+**
**Wakeling et al.** **[**63**]** 2005, Europe		**+**	**+**	**+**	**+**	**+**
**Weineberg et al.** **[**64**]** 2017, Australia	**+**	**+**	**+**	**+**	**+**	**+**
**Weineberg et al.** **[**65**]** 2019, Australia	**+**	**+**	**+**	**+**	**+**	**+**
**Wilson et al.** **[**66**]** 1999, Europe	**+**	**+**	**+**	**+**	**+**	
**Wu et al.** **[**67**]** 2017, China	**−**	**−**	**−**			
**Zhang el al** **[**68**].** 2013, China		**+**	**+**		**+**	**+**
**Zheng et al.** **[**69**]** 2013, China	**+**	**+**	**+**	**+**	**+**	**+**

This is a two-part tool, addressing seven specific domains (namely sequence generation, allocation concealment, blinding of participants and personnel, blinding of outcome assessment, incomplete outcome data, selective outcome reporting, and ‘other issues’) that are strongly associated with bias reduction. The green plus indicates low risk of bias, the red minus indicates high risk of bias, the white color indicates unclear risk of bias

### Quantitative data synthesis

**Fig. 2:**
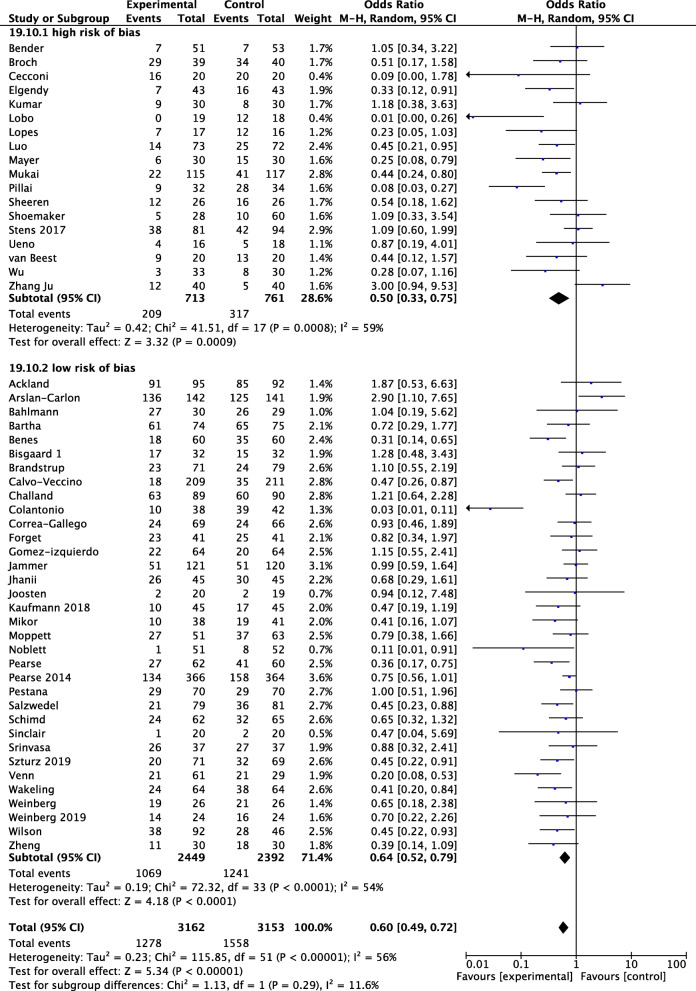
Rates of postoperative complications in subgroups defined according to risk of bias (see text for details) with odds ratios (ORs) and 95% confidence intervals (CI). The pooled OR and 95% CI are shown as the total. The size of the box at the point estimate of the OR gives a visual representation of the “weighting” of the study. The diamonds represent the point estimate of the pooled ORs and the length of the diamonds is proportional to the CI

In 52 RCTs, 6325 patients were enrolled. Of these, 3162 were randomized to perioperative GDT and 3153 were randomized to control. In the overall population, 2836 patients developed at least one complication: 1278 (40%) were randomized to perioperative GDT, and 1558 (49%) were randomized to control. Pooled OR was 0.60 and 95% CI was 0.49–0.72 (Fig. 2). The sensitivity analysis showed that the significant effect of GDT on postoperative complications was confirmed by low risk of bias RCTs, with high statistical heterogeneity and inconsistency (OR 0.64, 95% CI 0.52–0.79, *p* < 0.00001, *Q* statistic *p* = 0.0001; *I*^2^ = 56 %, 34 RCTs, 4841 patients) (Fig. 2).

**Fig. 3:**
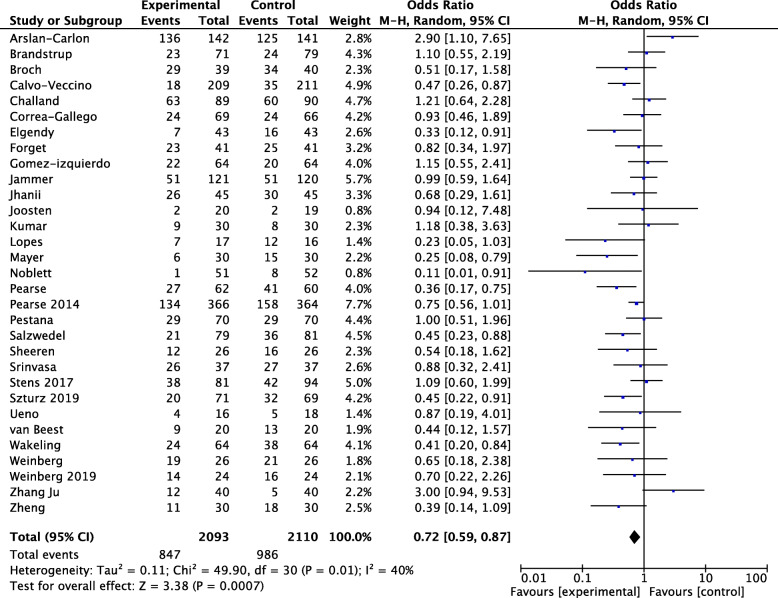
Rates of postoperative complications in patients undergoing abdominal surgery, with odds ratios (ORs) and 95% confidence intervals (CI). The pooled OR and 95% CI are shown as the total. The size of the box at the point estimate of the OR gives a visual representation of the “weighting” of the study. The diamonds represent the point estimate of the pooled ORs and the length of the diamonds is proportional to the CI

**Fig. 4:**
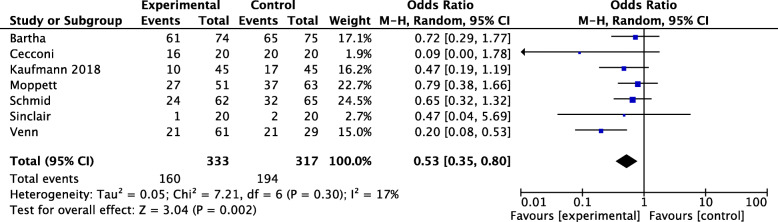
Rates of postoperative complications in patients undergoing orthopedic surgery, with odds ratios (ORs) and 95% confidence intervals (CI). The pooled OR and 95% CI are shown as the total. The size of the box at the point estimate of the OR gives a visual representation of the “weighting” of the study. The diamonds represent the point estimate of the pooled ORs and the length of the diamonds is proportional to the CI

**Fig. 5:**
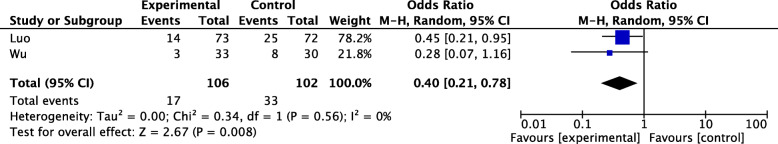
Rates of postoperative complications in patients undergoing neurosurgery, with Odds Ratios (ORs) and 95% confidence intervals (CI). The pooled OR and 95% CI are shown as the total. The size of the box at the point estimate of the OR gives a visual representation of the “weighting” of the study. The diamonds represent the point estimate of the pooled ORs and the length of the diamonds is proportional to the CI

The subgroup analysis enrolling major abdominal patients showed a significant result (OR 0.72, 95% CI 0.59–0.87, *p* = 0.0007, *Q* statistic *p* = 0.01, *I*^2^ = 40%, 31 RCTs, 4203 patients) (Fig. 3). A significant effect was observed in those RCTs enrolling exclusively orthopedic procedures (OR 0.53, 95% CI 0.35–0.80, *p* = 0.002, *Q* statistic *p* = 0.30; *I*^2^ = 17%, 7 RCTs, 650 patients) (Fig. 4). Also, neurosurgical procedures seemed to benefit from GDT (OR 0.40, 95% CI 0.21–0.78, *p* = 0.008, *Q* statistic *p* = 0.56; *I*^2^ = 0%, 2 RCTs, 208 patients, Fig. 5). Only 2 RCTs considered exclusively vascular surgery, and the pooled OR showed a non-significant effect of GDT on postoperative complications (OR 1.18, 95% CI 0.56–2.46, *p* = 0.67, *Q* statistic *p* = 0.79; *I*^2^ = 0%, 2 RCTs, 168 patients) as well as for thoracic surgery (OR 1.04, 95% CI 0.28–3.88, *p* = 0.95, *Q* statistic *p* = 0.01; *I*^2^ = 77%, 3 RCTs, 371 patients) (Supplementary material). For other surgeries, no other subgroup analyses were performed due to the very low number of RCTs included.

A strategy adopting only fluids yielded significant results (OR 0.67, 95% CI 0.47–0.97, *p* = 0.04, 17 RCTs, 1937 patients), as well as one using fluids and inotropes (OR 0.563, 95% CI 0.45–0.70, *p* < 0.00001, 35 RCTs, 4378 patients); both analyses had high statistical heterogeneity (Table 3). In both analyses, abdominal procedures were the most frequent ones. Considering only major abdominal surgery, using fluids alone yielded not significant results (OR 0.87, 95% CI 0.64–1.19 *p* = 0.39, *Q* statistic *p* =0.07; *I*^2^ = 40%, 13 RCTs, 1627 patients), while adopting a combined strategy with fluids and inotropes showed significant results (OR 0.63, 95% CI 0.49–0.79 *p* = 0.0001, *Q* statistic *p* = 0.09; *I*^2^ = 32%, 18 RCTs, 2476 patients). Also, in orthopedic surgery, a GDT strategy adopting only fluids yielded not significant result (OR 0.43, 95% CI 0.15–1.22 *p* = 0.11, *Q* statistic *p* = 0.009; *I*^2^ = 59%, 3 RCTs, 242 patients), while a strategy adopting fluids and inotropes showed significant results (OR 0.59, 95% CI 0.37–0.94 *p* = 0.03, *Q* statistic *p* = 0.56; *I*^2^ = 0%, 4 RCTs, 406 patients). No further analyses were possible in other kind of surgeries.

**Table 3:** The results of the subgroup analyses. RCTs were divided considering the kind of surgery (i.e., major abdominal, orthopedic, vascular, and so on) and on the basis of the strategy adopted (i.e., only fluids or fluids and inotropes)

Patients with complications All studies	n. of RCTs (references)	Treatment *n*/*N*	Control *n*/*N*	OR (95%CI)	*p* value	*I*^2^	*q* statistic *p* value
Fluids	17 (20, 26, 29, 31, 33–35, 37, 41, 45, 47, 51, 53, 56, 57, 62, 63)	472/976	520/961	0.67 (0.47–0.97)	0.04	63%	0.0003
Fluids and inotropes	35 (5, 21–25, 22, 28, 30, 32, 36, 38–40, 42–44, 46, 48–50, 52, 54, 55, 58–61, 64–69)	806/2186	1038/2192	0.56 (0.45–0.70)	< 0.00001	50%	0.0004
Major abdominal surgery							
Fluids	13 (20, 26, 29, 31, 33–35, 37, 41, 47, 53, 57, 63)	414/812	432/815	0.87 (0.64–1.119)	0.39	40%	0.07
Fluids and inotropes	18 (5, 27, 32, 36, 39, 43, 48–50, 52, 58–61, 64, 65, 68, 69)	43371281	554/1285	0.63 (0.49–0.79)	< 0.0001	32%	0.09
Orthopedic surgery							
Fluids	3 (45, 56, 62)	49/132	60/112	0.43 (0.15–1.122)	0.11	59%	0.09
Fluids and inotropes	4 (22, 28, 38, 54)	11/201	134/205	0.59 (0.37–0.94)	0.03	0%	0.56

*OR* odds ratio, *CI* confidence interval, *RCT* randomized controlled trial

In those RCTs adopting only fluids as optimization strategy, patients in the GDT group received more colloids (Table 4) and less crystalloids (Table 4) than patients in the control group. The total volume of fluid was not significantly different between the GDT and the control group.

**Table 4:** Total amount of fluids, colloids, and crystalloids used in all RCTS included

Patients with complications All studies	n. of study (references)	Treatment	Control	Standard mean difference (95%CI)	*p* value	*I*^2^	*q* statistic *p* value
Total fluids (ml)	9 (22, 31, 33, 34, 37, 41, 43, 46, 53)	473	484	− 1.14 (− 2.38,0.11)	0.07	98%	*p* < 0.00001
Colloids (ml)	12 (20–22, 26, 29, 33, 34, 45–47, 53, 56)	796	820	0.71 (0.07, 1.36)	0.003	97%	*p* < 0.00001
Crystalloids (ml)	12 (20–22, 26, 29, 33, 34, 37, 45–47, 56)	765	786	− 2.07(− 1.03, − 3.11)	0.0001	99%	*p* < 0.00001

*OR* odds ratio, *CI* confidence interval, *RCT* randomized controlled trial

## Discussion

The epresent meta-analysis suggests that GDT can significantly reduce postoperative complications. This effect is confirmed when only low risk of bias RCTs were included in the analysis. The surgical procedures that seem to benefit most are abdominal, orthopedic, and neurosurgical ones.

GDTe was initially proposed for the maintenance of an optimal cardiac output, in order to allow prompt restoration of perfusion and avoid cellular hypoxia and tissue injury [55]. Nowadays, GDT does not aim to a maximized cardiac output but rather pursues personalized hemodynamic management assessing blood flow and fluid responsiveness, in order to prevent not only tissue hypoperfusion and hypovolemia, but also perioperative fluid overload, since both are associated with adverse postoperative outcomes [70, 71].

Several RCTs and meta-analyses show that GDT reduces postoperative complications in high-risk surgical patients, regardless the monitoring or the target [49, 72]. Therefore, the use of GDT has been suggested from expert groups [73, 74], at least in high-risk patients and in major abdominal surgery, when high intravascular volume replacement is needed. However, the great heterogeneity of the studies exploring GDT effects, in terms of types of surgery, timing, type of monitoring device, the hemodynamic variables assessed and targeted and the types and amounts of fluids, vasopressors, and/or inotropes used can not be ignored [75], and make a definite conclusion on GDT application much less clear. Focusing on specific type of procedures or strategies could add more clarity to the available evidences.

The incidence of postoperative complications is well documented in abdominal surgery (from 12% after hepatectomy to 44% after esophagectomy) [69], and similar data are reported in other type of surgical procedures: for example, in fracture surgery the incidence of postoperative complications ranges from 7 to 42% [76]. Also, vascular surgery shows similar trends, with a range varying from 21 to 33% [69]. The incidence of systemic complications in neurosurgical procedures is estimated approximately at around 14% [77].

Our results confirm the significant reduction of postoperative complications in major abdominal surgery. Differently from others [77, 78], however, the present meta-analysis yielded significant results also in other kind of surgeries, suggesting that GDT application could be extended to other surgical settings, since also orthopedic and neurosurgical procedures can benefit from a GDT approach, while no effects were seen in thoracic or vascular surgery. Moreover, considering all types of surgeries, a GDT approach that uses only fluids or fluids and inotropes has shown significant results, while in major abdominal and orthopaedic surgery, only a strategy adopting inotropes in addiction to fluids yielded significant results. It is possible to argue that GDT, guiding to an individualized and timely fluid administration, allows to use fluids judiciously when they are needed, but also to avoid unnecessary fluid loading when hemodynamic targets are already met [6, 76]. This strategy can allow to avoid fluid overload from one side and maintain tissue perfusion on the other, thus reducing postoperative complications. When fluids are not sufficient, a combination of vasoconstrictors to maintain an adequate mean arterial pressure and of inotropes to increase stroke volume, guided by advanced hemodynamic monitoring could help to assure adequate perfusion [73, 74]. The present results suggest that in those surgical settings expected to be managed with large amounts of fluids or enrolling old, high-risk patients, such as abdominal or orthopedic ones, a GDT approach including fluids and inotropes is effective in reducing postoperative complications. We cannot state if the effects of fluids and inotropes are synergistic or the beneficial effect of one intervention counteracts the adverse effect of the other, but it can be supposed that a more extensive hemodynamic monitoring and targeting can help to guide perioperative management and to reduce postoperative complications in these specific surgical scenarios. In this way, for example, patients with a reduced physiologic reserve may benefit of additional and early administration of inotropic drugs to increase oxygen delivery and counteract hypoperfusion. The low number of patients involved, the mixed nature of surgical procedures and the lack of individual data are all possible explanations to the inconclusive findings in the other surgical procedures (thoracic or vascular surgery).

Another finding of our meta-analysis is that the total volume of fluids did not increase with the use of GDT. Patients received more colloids, but less crystalloids, so that the total volume of fluids was not significantly different between the control and the GDT group. This finding goes against the perception or the fear that using hemodynamic optimization protocols may be associated with excessive fluid administration, but, on the contrary, supports the idea that GDT helps clinicians to give the right amount of fluid to the right patients at the right time.

A major limitation of our analysis is the presence of heterogeneity in defining postoperative complications, and keeping this in mind a random effects model was used even when the estimated amount of heterogeneity was low. A high heterogeneity was found in almost all subgroups, reducing the strength of the results. Moreover, even if we tried to control clinical heterogeneity with subgroup analyses splitting studies on the basis of surgery type and targets, statistical heterogeneity remained high, and therefore, the results should be interpreted with caution. Third, the consistency of data reporting postoperative fluid administration is lacking, as well as data on oral fluid intake and perioperative management is missing in many studies, so direct comparison is difficult. Finally, the definition of postoperative complications is another crucial point of all these studies. We choose to consider the rate of patients who had at least one complication, like other authors proposed [6] since the evaluation of specific organ-related events has numerous bias linked to the definition of postoperative event, the overlapping of postoperative complications and the risk to over-estimate the total number of complications.

## Conclusion

The present meta-analysis, within the limits of the existing data, the clinical and statistical heterogeneity, gives new suggestions on the beneficial effect of GDT in reducing postoperative morbidity rate in other type of surgeries, different from the major abdominal. These results call for other RCTs with the aim to explore the real impact of hemodynamic goal-directed strategy and its specific issues (i.e., monitoring tools and targets, means adopted, patients to enroll) in different surgical settings.

## Supplementary Information

**Additional file 1: Supplementary file 1.** The search strategies used for the MEDLINE, The Cochrane Library and EMBASE databases.

## Data Availability

The datasets used and/or analyzed during the current study are available from the corresponding author on reasonable request.

## Abbreviations

GDT: Goal-directed therapy
ReCTs: Randomized controlled trials
POSSUM score: Physiological and Operative Severity Score for the enUmeration of Mortality and morbidity
ASA: American Society of Anestesiology
ORs: Odds ratios
CIs: 95% confidence intervals
WMD: Weighted mean difference
