# Supplementary figures and images for: Perioperative goal-directed therapy and postoperative complications in different kind of surgical procedures: an updated meta-analysis
Source: J Anesth Analg Crit Care. 2021 Dec 15;1:26. doi: 10.1186/s44158-021-00026-3 (PMC10245481; doi:10.1186/s44158-021-00026-3)

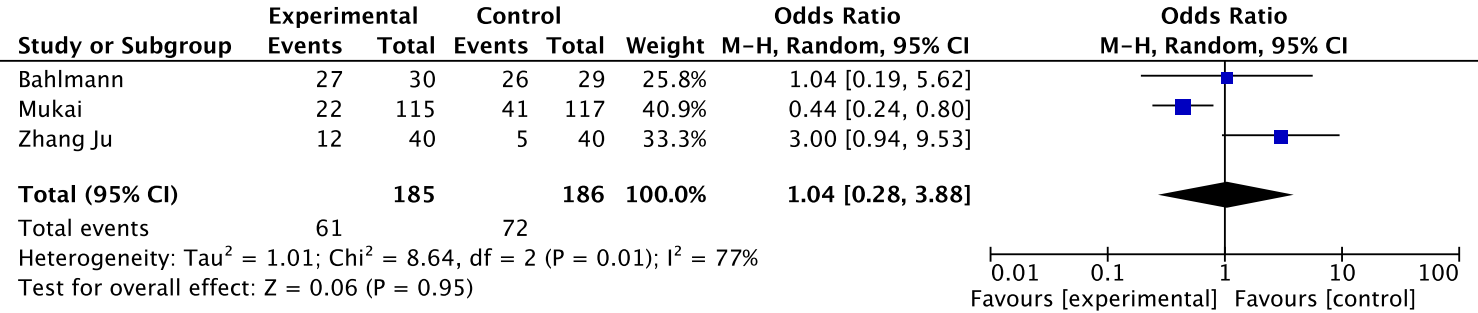

Supplement: Supplementary file 1 — Additional file 1: Supplementary file 1. The search strategies used for the MEDLINE, The Cochrane Library and EMBASE databases. [file 44158_2021_26_MOESM1_ESM.pdf]
